# Analysis of Clinical, Immunological and Molecular Features of Leukocyte Adhesion Deficiency Type I in Egyptian Children

**DOI:** 10.1007/s10875-024-01693-x

**Published:** 2024-04-05

**Authors:** Mai Magdy Saad, Radwa Alkady, Alia Eldash, Rabab E. El Hawary, Safa S. Meshaal, Nermeen M. Galal, Aisha M. Elmarsafy

**Affiliations:** 1 Pediatrics Department, Faculty of Medicine, Cairo University, Cairo, 11562 Egypt; 2 Clinical Pathology Department, Faculty of Medicine, Cairo University, Cairo, Egypt

**Keywords:** Genetic diagnosis, Inborn error of immunity, Leukocyte adhesion deficiency, Phagocytic defect

## Abstract

**Purpose:**

Leukocyte adhesion deficiency (LAD) represents a rare group of inherited inborn errors of immunity (IEI) characterized by bacterial infections, delayed umbilical stump separation, and autoimmunity. This single-center study aimed at describing the clinical, immunological, and molecular characterizations of 34 LAD-I Egyptian pediatric patients.

**Methods:**

Details of 34 patients’ personal medical history, clinical and laboratory findings were recorded; Genetic material from 28 patients was studied. Mutational analysis was done by Sanger sequencing.

**Results:**

Omphalitis, skin and soft tissue infections with poorly healing ulcers, delayed falling of the umbilical stump, and recurrent or un-resolving pneumonia were the most common presentations, followed by chronic otitis media, enteropathy, periodontitis; and recurrent oral thrush. Persistent leukocytosis and neutrophilia were reported in all patients, as well as CD18 and CD11b deficiency. CD18 expression was < 2% in around 90% of patients. Sixteen different pathological gene variants were detected in 28 patients who underwent *ITGß2* gene sequencing, of those, ten were novel and six were previously reported. Three families received a prenatal diagnosis. Patients were on antimicrobials according to culture’s results whenever available, and on prophylactic Trimethoprim-Sulfamethoxazole 5 mg/kg once daily, with regular clinical follow up. Hematopoietic stem cell transplantation (HSCT) was offered for 4 patients. However due to severity of the disease and delay in diagnosis, 58% of the patients passed away in the first 2 years of life.

**Conclusion:**

This study highlights the importance of early diagnosis and distribution of *ITGß2* gene mutation in Egyptian children. Further molecular studies, however, remain a challenging necessity for better disease characterization in the region.

## Introduction

Leukocyte adhesion deficiency (LAD) represents a rare group of inherited errors of immunity (IEI) that is characterized by the absence of adhesion molecules on leukocytes, preventing them from migrating to the site of infection/inflammation. Accordingly, individuals with LAD frequently develop leukocytosis, early onset indolent bacterial infections, delayed umbilical stump separation, as well as autoimmunity [1]. Based on the site of defect throughout the activation cascade, four different LAD types have been reported, LAD I- IV [2]. Based on CD18 expression on neutrophils, LAD-I was sub-classified into severe: < 2% (LAD-I °); moderate: 2–30% (LAD-I ^−^); and mild: ≥ 30% (LAD-I^+^) [3]. Mutations in the *ITGß2* gene encoding for the β 2-integrin component (CD18) cause LAD-I disease, for which hematopoietic stem cell transplantation (HSCT) remains the main curative treatment [4]. In this study, we describe the clinical presentations, laboratory findings, molecular diagnosis, follow-up records and outcomes of 34 Egyptian LAD-I patients, diagnosed and following up at the Pediatric Primary Immunodeficiency Unit (PPIU) in Cairo University Specialized Children’s Hospital.

## Patients and Methods

Our study included 34 Egyptian patients with LAD-I phenotype evaluated at PPIU between 2009 and 2023. Informed consents were obtained from parents or legal guardians and the study was approved by the local institutional review board. Details of each patient’s personal medical history, clinical and laboratory findings were recorded, and LAD-I was diagnosed according to the International Union of Immunodeficiencies (IUIS) criteria for IEI [5] and the European Society for Immunodeficiencies (ESID) criteria. Genetic material was available for only 28 patients.

### Immunologic Analyses

Blood samples (3 ml) were drawn in ethylenediamine tetra acetic acid (EDTA). Peripheral blood neutrophils’ integrin expression was analyzed using flow cytometry. Fluorochrome-labeled monoclonal antibodies (mAbs) against CD18 PE (BC M1570U) and CD 11b FITC (BC IM0530U) were utilized for staining as previously described [6]. All samples were analyzed on an FC500 flow cytometer (Beckman Coulter) or FACS Canto-II flow cytometer (BD).

### Genetic Analysis

Genomic DNA was extracted using a QIAamp DNA blood mini kit, according to the manufacturer’s instructions. *ITGß2* gene was sequenced as previously described using primers designed by Yassaee VR et al. [7], except for exon 13 primers used according to Mortezaee FT et al. [8]. Polymerase chain reaction (PCR( products were sequenced on a (3500 Genetic Analyzer Applied Biosystems, USA) using the same primers that were used to amplify the PCR fragments. Sequences were compared with the reference sequence published by the National Centre for Biotechnology Information (NCBI) (*ITGß2*; NM_000211.5) and were analyzed using the Basic Local Alignment Search Tool (BLAST). The identified variants were categorized as pathogenic/likely pathogenic/variant of uncertain significance (VUS)/likely benign/benign based on American College of Medical Genetics (ACMG) guidelines [9].

## Results

We included 34 LAD-I patients (18 females and 16 males) born to 29 known consanguineous families, and only 4 families not known to be consanguineous. The age at onset of manifestations ranged between 3 days to 7 months, (median 0.6 months), while the age at diagnosis ranged between 1 and 84 months (median 4 months). There was a diagnostic delay that ranged between 0 and 83 months (median 3.35 months). P26, however, symptomatized a bit later (24 months), and presented to be diagnosed at the age of 12 years. Fifteen families had prior sib’s passing away after a history highly suggestive of LAD disease, while only 1 family presented with their first offspring diagnosed as LAD-I (P33) followed by a second-born (P34) similarly affected sib.

### Clinical Findings

Seventeen patients (50%) were underweight with their weight below the 5th centile. The most common presenting features (85.3%) were umbilical stump disease (delayed falling of the umbilical stump &/or omphalitis), skin and soft tissue disease (including poorly healing ulcers, infections, rash, pyoderma gangrenosum, and perianal thrush). This was followed by recurrent/unresolving pneumonia (41%), chronic otitis media (35.2%), enteropathy (29.4%), and oral thrush (17.6%). Other less common infections included blepharitis, dactylitis, orbital cellulitis, peritonitis, and meningitis (*n* = 1 each). Periodontitis and premature loss of teeth, as well as nail dystrophy, were each found in 4 patients with ages between 4 and 21 years at the time of the study. Patients’ demographic and clinical data are summarized in Table 1.

**Table 1:** Demographic and clinical features of the reported LAD-I patients

Patient’s No	Gender	Consanguinity	Affected Sibs	Age at onset (m)	Age at diagnosis (m)	Delay in diagnosis (m)	Umbilical stump disease	Skin and soft tissue disease	Other Clinical presentations	*Outcome
**P1**	♂	-	-	0.3	6	5.7	Yes	Yes	FTT, pneumonia, OM, oral thrush, enteropathy, periodontitis	died (8y)
**P2**	♀	+	-	1.5	3	1.5	Yes	Yes	FTT, splenomegaly	lost to follow up
**P3**	♂	+	-	1.5	36	34.5	Yes	Yes	FTT, OM, enteropathy	HSCT was done at 3 y lost to follow up 2 y after
**P4**	♀	+	-	0.3	2.5	2.2	Yes	Yes	FTT	lost to follow up
**P5**	♂	-	-	0.8	3	2.2	Yes	Yes	FTT, orbital cellulitis	died (6 m)
**P6**	♂	+	1 ♂ (died-1 m-sepsis) 1 ♀ (died-1 m-sepsis)	2	4	2	Yes	-	FTT, OM, oral thrush, peritonitis	died (1y)
**P7**	♂	+	1♂ (died-4 m-sepsis)	1	60	59	No	Yes	FTT, pneumonia	lost to follow up
**P8**	♂	-	-	0.5	10	9.5	Yes	Yes	meningitis, OM	alive (2y)
**P9**	♀	+	-	0.4	1	0.6	Yes	-	-	died (1 m)
**P10**	♀	+	1♂ (died-43d-omphalitis/sepsis)	0.7	3	2.3	Yes	Yes	FTT, pneumonia, enteropathy, oral thrush	alive (2y)
**P11**	♂	+	1♀ (died-4y-mastoiditis/sepsis)	0	13	13	Yes	Yes	pneumonia, OM	died (1.5y)
**P12**	♀	+	1♀ (died-6y-infections/sepsis)	4	36	32	Yes	Yes	pneumonia, OM	lost to follow up
**P13**	♂	+	-	1	4	3	Yes	Yes	FTT, pneumonia, OM, oral thrush, enteropathy, dactylitis, periodontitis, nail dystrophy, convulsions	died (4y)
**P14**	♀	+	2 ♀ (died-2&10 m-omphalitis/sepsis)	1	10	9	Yes	Yes	blepharitis	HSCT done at 16 m alive (5y)
**P15**	♀	+	1 ♂ (died-7d-sepsis) 1 ♀ ( died-2y-skin infections-OM- omphalitis/sepsis)	0.3	8	7.7	Yes	Yes	OM	died (8 m)
**P16**	♂	+	♀ (died-3 m-omphalitis/sepsis)	0.5	2	1.5	Yes	Yes	OM	died (3.5 y)
**P17**	♂	+	1 ♀ (died-2.5y-pneumonia)	0.1	1	0.9	Yes	Yes	-	HSCT done at 4 m alive (5y)
**P18**	♀	+	1♀ (died-2 m-sepsis)	0.5	8	7.5	Yes	Yes	FTT, enteropathy	died (1y)
**P19**	♂	+	1 ♀ (died-7 m-pneumonia)	1	2	1	Yes	-	pneumonia, Oral thrush	lost to follow up
**P20**	♀	-	-	1.5	2	0.5	Yes	-	-	died (1.5y)
**P21**	♂	+	-	5	15	10	No	Yes	FTT, pneumonia, OM, splenomegaly	alive (3y)
**P22**	♂	+	1♀ (died-2 m-sepsis)	0.3	2	1.7	Yes	-	hepatomegaly	died (3 m)
**P23**	♂	+	-	0.3	1	0.7	Yes	Yes	FTT, enteropathy	alive (3y)
**P24**	♀	+	-	0.1	5	4.9	Yes	Yes	FTT, pneumonia	died (1y)
**P25**	♀	+	-	-	48	-	Yes	Yes	-	lost to follow up
**P26**	♀	+	1♂ (Skin infections- HSCT at 9y- GVHD - died-9y-) 1♀ (Died-1y- sepsis)	24	144	120	No	Yes	-	alive (21y)
**P27**	♀	+	-	0.3	4	3.7	Yes	Yes	enteropathy	HSCT done at 10 m alive (5y)
**P28**	♀	+	1 ♀ (died-1 m-sepsis)	0.4	2	1.6	Yes	Yes	oral thrush, enteropathy	died (6 m )
**P29**	♀	+	-	0.5	3	2.5	No	Yes	FTT, OM, pneumonia, periodontitis.	alive (7y)
**P30**	♂	+	-	1	84	83	Yes	Yes	Periodontitis	alive ( 21y)
**P31**	♀	+	-	1	7	6	Yes	Yes	FTT, pneumonia, OM, enteropathy	alive (2y)
**P32**	♂	+	1 ♂ (died-45d-omphalitis/sepsis)	0.1	4	3.9	Yes	Yes	pneumonia, enteropathy, splenomegaly	lost to follow up
**P33#**	♀	+	♂ & ♀ twins (died-7d-sepsis) 1♀ (P 34)	7	14	7	No	Yes	FTT, pneumonia, HSM	died (15 m)
**P34#**	♀	+	♂ & ♀ twins (died-7d-sepsis) 1♀ (P 33)	3	3	0	Yes	Yes	FTT	died (5 m)

m = months; y = years; d = days; OM = otitis media; FTT = failure to thrive; HSM = hepatosplenomegaly; GVHD: Graft versus host disease

*Ages mentioned in outcome were either the living patients’ ages at the time of the study, or the age at which they deceased

# Siblings

### Laboratory Data

**Fig. 1:**
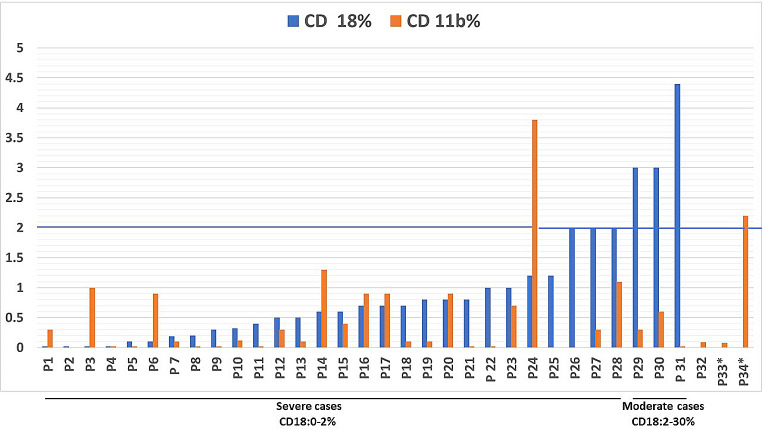
Distribution of CD18 and CD11b among LAD I patients

Naturally, all patients had leukocytosis, (mean total leucocytic count 62.5 ± 25 × 10^3^/dl), as well as neutrophilia (mean absolute neutrophilic count 47 ± 23.3 × 10^3^/dl); mean absolute lymphocytic count was 13 ± 7.1 × 10^3^/dl. Most of the patients (88.5%) were anemic, (mean Hb level 8.4 mg/dl) and 10 of them required recurrent transfusions. By definition, CD18 and CD11b were deficient in all patients (0-4.4%, mean 0.9%; and 0-3.8%, mean 0.53%, respectively). Most of the patients (90.2%) had severe CD18 deficiency (< 2% expression), while a few others showed moderate deficiency, and there were no patients with mild deficiency (Fig. 1). *Pseudomonas aeruginosa* was the most commonly isolated organism from blood, wound & ear swabs, and urine cultures in this cohort, followed by Methicillin-resistant Staphylococcus aureus (*MRSA)*, *Klebsiella*, *Proteus*, *Corynebacteria*, *E coli*, *Enterococci*, and *Candida* albicans.

### Molecular Studies

In the current study, direct Sanger sequencing of the *ITGß2* gene was performed for 28 patients. Sixteen different gene variants were detected; of which ten were novel variants and six were previously reported. All patients showed homozygous phenotype except P1 (non-consanguineous parents) who showed a compound heterozygous phenotype (p.Gly284Ser and p.Glu668Glyfs*23). The most affected exon was exon 7; (2 variants in 11 patients).

Eight missense variants were detected in 20 patients: p.Gly284Ser (rs137852616) (10 patients); p.Gly169Arg (rs137852612) (3 patients); p.Cys62Tyr (2 patients); p.Gly167Val; p. Gly273Arg (rs137852618); p.Pro302Leu (rs750067657); p.Gly555Arg; and p.Cys557Tyr (in 1 patient each). Four deletion variants were detected in 1 patient each: p.Gly40Alafs*7; p.Val103*; p.Gly617Alafs*16; and p.Glu668Glyfs*23. Three nonsense mutations were detected in one patient each: p.Arg188* (rs148877937); p.Gln218*; and p.Cys459*. One insertion variant was detected in one patient: p.Val103Serfs*39. Ten of our patients’ variants were previously reported by our center (P4, P5, P11, P14, P15, P18, P19, P23, P24, and P28) [10].

Prenatal diagnosis (PND) was offered to 3 families with previously diagnosed index cases (family of P5 done twice, family of P10 and P23 once), via chorionic villus sampling (CVS) or amniotic fluid sampling (AFS) performed in the 12th week of gestation as previously described [11]. The first unborn sibling of P5 was identified as a carrier for the pathogenic variant p.Gly169Arg, while the other fetus a year later showed a homozygous wild-type phenotype. P23’s unborn sibling showed a homozygous wild-type phenotype. AFS was done for the unborn sibling of P10 and was found to have a homozygous pathogenic variant p.G284S. The pregnancies with normal or carrier fetuses were continued, while those with diseased fetuses were dealt with according to the families’ decisions after proper counseling.

### Patients’ Outcome

58% of the patients succumbed in the first 2 years of life except for P1, P13, and P16 (died at the age of 8, 4, and 3.5 years respectively), mostly due to sepsis complicating omphalitis/peritonitis, pneumonia, skin and soft tissue disease, and orbital cellulitis. One patient died due to severe enteropathy. HSCT was offered to 4 patients (P3, P14, P17, and P27) from matched, related donors with successful engraftments. P17 transplanted at 4 months experienced recurrent otitis media complicated with mastoiditis, and P3 transplanted at 3 years developed GVHD 1 year later and was treated with steroids and immunosuppressives.

## Discussion

88% of our patients were born to consanguineous families, hence highlighting the burden of autosomal recessive IEI in highly consanguineous populations like Egypt, Pakistan, and Israel [12, 13]. Although a family history of sibs with a typical LAD-I clinical phenotype was reported in 49% of the study population, only 2 of those index cases/sibs presented to be diagnosed as LAD-I before their demise, and this was comparable to other studies [3, 13].

97% of the patients presented in their first year of life (except for P26). The delay in diagnosis almost halved in the last five years, with a median of 2.3 months. This reflects the improved awareness of many developing countries’ pediatricians, as currently witnessed in Egypt and Pakistan [13]. Patient 26 and two of her siblings initially symptomatized around 2 years of age with a mild phenotype (recurrent skin rash and ulcers, yet thrived well); they presented for diagnosis at the ages of 12, 5 and 13 years, respectively. The same family had a sib manifesting and died before the age of 1 year with a stormy course of recurrent infections and sepsis. One of the siblings received a fully matched related HSCT at the age of 9 years, which was unfortunately complicated by GVHD, resulting in his loss. P30 presented for diagnosis at the age of 7 years, although his symptoms started at the age of 1 month. The diagnosis was also reached at an older age in P3, P7, P12, and P25 (at the ages of 3, 5, 3 and 4 years respectively), despite early symptoms in the first few months of life. **Celiksoy et al.**. described three Turkish siblings with a very similar presentation who were diagnosed with LAD-I in their early adolescence [14].

Umbilical stump disease and/or skin and soft tissue disease were the first presenting symptoms in all patients included in this study, among many other studies [3, 13–18, 20–23].

Anecdotally delayed umbilical separation, however, is not a hallmark of the disease, while umbilical stump disease are a similar conclusion reported in other studies [12, 15, 19].

Pneumonia, chronic otitis media, enteropathy, and oral thrush were also common symptoms presenting throughout the disease course [3, 15].

In patients surviving to older ages, autoinflammatory symptoms prevailed in the form of periodontitis and loss of teeth, as well as enteropathy. This was evident in several studies recognising periodontal disease as the most common manifestation in surviving older LAD-I patients without HSCT [24, 25]. The introduction of Ustekinumab for those patients at the same dose used in patients with psoriasis yielded promising outcomes in the resolution of persistant oral inflammation and extensive sacral wounds. Although Ustekinumab is effective in early complete blocking of interleukin-17, prolonged therapy is recommended in patients with periodontitis brought on by the ongoing inflammatory process [26]. Unfortunately, this modality of treatment is not yet conducted in our center due to financial restraints.

The scope of opportunistic, aggressive organisms isolated from our patients did not differ from the ones described in the literature, owing to the chronic inflammatory nature of the skin and soft tissue lesions [15, 18, 27, 28].

As has been reported in similar studies, LAD-I^0^ was also the predominant phenotype in our population [1, 3, 22, 29, 30]. All LAD-I^0^ had severe manifestations early in life, and 65% of the ones following in our clinic lost their lives. Only 3 patients were LAD-I^−^, and their CD18 level was barely above 2% (P28, P29, and P30). All of them are alive, however, suffering from aggressive autoinflammatory symptoms in the form of periodontitis and enteropathy [4, 25, 26].

Anemia in LAD-I patients is a well-documented finding (prevalent as high as 67%, 72%, and 85% of patients in **Wolach et al.**, **Yaz et al.**, as well as our cohort, respectively), which can be attributed to chronic inflammation and poor intake due to recurrent infections and frequent hospitalization [12, 15].

58% of the study population perished. This is sadly comprehensive in our setting, given the severity of the disease and the limited national access to a timely, curative HSCT. Comparable bleak outcomes can be found in a Spanish American study in 2018; an Indian and a Pakistani study in 2020, representing the morbidity and mortality burden associated with LAD-I disease. In Ankara, **Bakhtiar et al.** reported 3-year survival estimates of 84% in a cohort of 69 LAD-I patients following HSCT. The best survival rates were found in patients with sibling(s), matched family, or unrelated donor transplants. GVHD was the major cause of death, followed by infections [3, 4, 13, 32].

*ITGß2* gene is located on chromosome 21q22.3 (OMIM *600,065), where a range of mutations have been identified in patients with LAD-I. A few mutations could cause a nonfunctional but normally expressed CD18 molecule. Most *ITGß2* mutations lead to reduced/null expression of β2-integrins on the leukocyte surface [33].

In our study, direct Sanger sequencing of the *ITGß2* gene revealed 16 disease-causing variants in 28 patients. These mutations were clustered mostly in exons 4, 6, 7, and 13. The frequency of the variant c.850G > A (p.Gly284Ser) was high in 10/28 patients (36%). This may reflect a founder effect due to high consanguinity and inbreeding in the Egyptian population, as previously reported in variants in other genes such as CYBA c.295_301del causing chronic granulomatous disease (CGD) [34].

About 43% (*n* = 7) of the variants were located in exon 5–9, a highly conserved region of the extracellular domain of CD18, followed by the cysteine-rich repeat region (CRR) domain (25%, *n* = 4). Similarly, **Roos et al.**, 2002 stated that most of the single nucleotide variations are found in a ~ 240-residue domain that is highly conserved in all β integrin subunits and encoded by exons 5–9 of *ITGß2* [35].

Out of the 16 identified disease-causing variants; 6 were previously reported, while the remaining 10 were novel and were not found in ExAC nor1000G suggesting heterogeneity in the mutation spectrum for LAD-I.

Two novel variants were detected in Exon 6 of the *ITGß2* gene: P14 with the c.500G > T variant, causing a change of amino acid sequence at position 167 from glycine to valine. Being 2 nucleotides away from the splice site might cause a splice site change and affect the protein features as this position is highly conserved. While P24 had a c.652 C > T variant causing severely truncated protein after 218 amino acids, the loss of protein function in this case can be due to nonsense-mediated mRNA decay [36].

Three novel variants were detected in Exon 4 of the ITGß2 gene; the missense variant c.185G > A was identified in P19 and P28, it caused a change of amino acid at position 62 from cysteine to tyrosine, which might lead to changes in the protein features. This variant has not previously been reported in LAD-I patients, although an adjacent missense variant c.184T > C; p.Cys62Arg was previously reported by Van De Vijver et al. in 2012 [37]. P18 showed c.306dupA causing a frameshift in the protein at position 103 from valine to serine and a truncation of the protein at amino acid 141. While P4 showed a homozygous deletion c.307delG causing a premature truncation of the protein at position 103.

Three novel variants were detected in Exon 13 of the *ITGß2* gene; The missense variant c.1663G > A found in P12, caused a change of amino acid at position 555 from glycine, which is a neutral nonpolar amino acid to arginine, which is a basic polar amino acid, this is predicted to lead to changes in the protein features. While P8 showed a homozygous missense mutation c.1670G > A, causing a change of amino acid sequence at position 557 from cysteine to tyrosine. Although this variant is not previously reported in LAD-I patients, another missense variant in the same position (c.1670G > C, p.C557S) was previously reported in van de Vijver et al., 2012 [37].

P17 showed a homozygous deletion c.1848delC causing a frameshift in the protein at position 617 from glycine to alanine and a truncation of the protein at amino acid 632. Loss of protein function in this case can either be due to protein truncation or nonsense-mediated mRNA decay.

P11 had a novel nonsense variant in exon 11 c.1377 C > A, causing a truncated protein after 459 amino acids, probably producing truncated mRNA, which was degraded through nonsense-mediated mRNA decay.

P1 showed heterozygous deletion in exon 14 c.2003_2004delAG causing a frameshift in the protein at position 668 from glutamate to glycine and a truncation of the protein at amino acid 690, as well as a heterozygous variant c.850G > A commonly found in our patients.

Identification of ten homozygous novel variants in the *ITGß2* gene in LAD-I patients of Egyptian origin not only expanded the mutation spectrum in the gene, but such findings will facilitate the screening of similar patients in the local population. Since the variants are segregated in an autosomal recessive manner, the findings will support genetic counselling for the families as well.

Four PND tests were offered to three families, and only one proved to have a homozygous pathogenic variant. PND services can enable early postpartum intervention in cases where families decide to proceed with the pregnancy that would result in an affected baby. It is worth mentioning that a similar approach was taken with other IEI disorders genetically diagnosed at our center.

## Conclusion

Though awareness of LAD-I diagnosis in Egypt has markedly improved recently, as evidenced by shortening in the delay of diagnosis, the death toll is still too high owing to the limited access to a timely HSCT. Further molecular studies, though still challenging, are essential to better characterize the disease genotype in the region, as more than half the detected mutations in our cohort were novel.

## Data Availability

No datasets were generated or analysed during the current study.
